# Supplementary material for: A substrateless, flexible, and water-resistant organic light-emitting diode
Source: Nat Commun. 2020 Dec 7;11:6250. doi: 10.1038/s41467-020-20016-3 (PMC7721873; doi:10.1038/s41467-020-20016-3)
Supplement: Supplementary file 1 — Supplementary Information [file 41467_2020_20016_MOESM1_ESM.pdf]

Supplementary Information

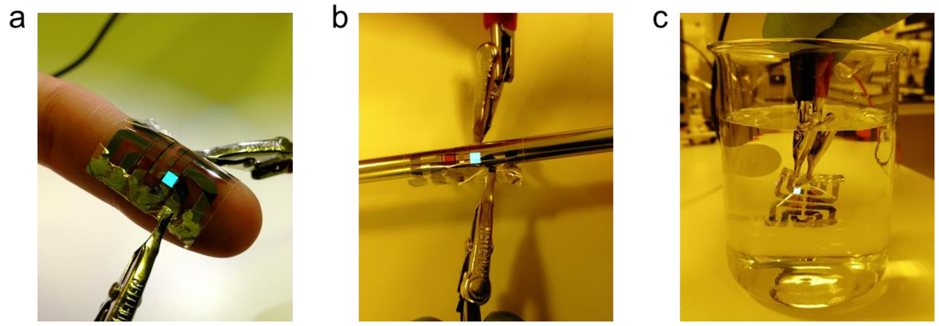

Supplementary Figure 1. Flexible, water-resistant fluorescent blue OLEDs. Photographs of a flexible blue OLED **a**, on a finger, **b**, wrapped around a screwdriver, and **c**, immersed in deionized water.

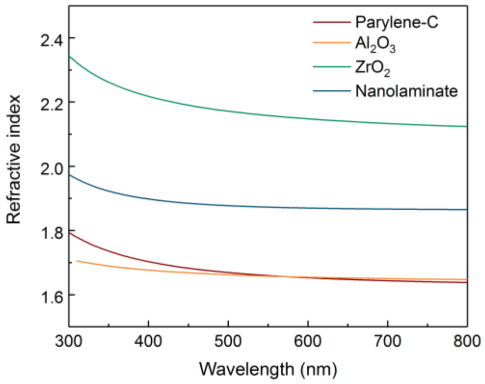

Supplementary Figure 2. Spectrally resolved refractive index of materials forming the flexible barriers. The refractive indices of parylene-C, Al_2O_3, ZrO_2, and the Al_2O_3/ZrO_2 nanolaminate were measured by variable angle spectroscopic ellipsometry. The nanolaminate shows an intermediate refractive index between Al_2O_3 and ZrO_2.

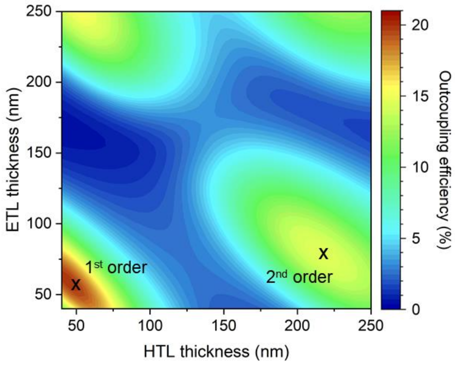

Supplementary Figure 3. Optical modelling of outcoupling efficiency as function of charge transport layer thickness. Outcoupling efficiency of OLEDs on a P/N/P lower barrier as function of HTL and ETL thicknesses, calculated using transfer matrix-based optical simulations. The 1st order maximum is reached for HTL and ETL thickness of 50 nm and 60 nm, respectively. A 2nd order maximum occurs at 220 nm (HTL) and 80 nm (ETL).

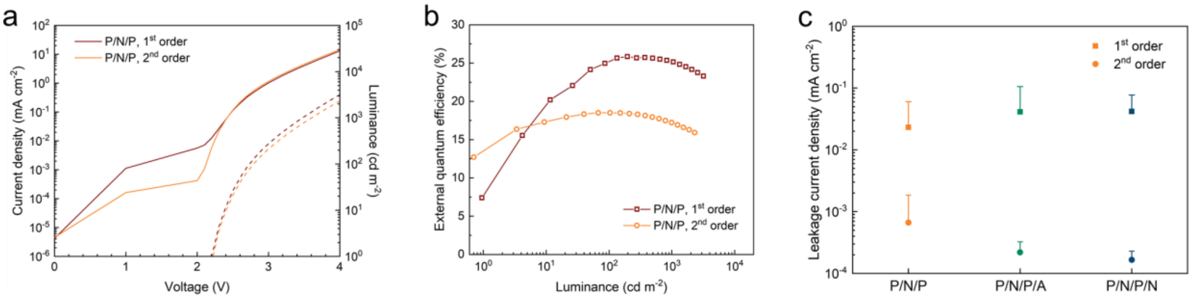

Supplementary Figure 4. Comparison of OLEDs with first and second order cavities. a, Current density (solid lines) and luminance (dashed lines) versus voltage for first and second order OLEDs with P/N/P lower barrier layer. **b**, External quantum efficiency for same devices, assuming Lambertian emission characteristics. **c**, Comparison of leakage current density of OLEDs with first and second cavity. Data points and error bars represent the average and standard deviation of five OLED pixels for each device type. Leakage current density is defined as the current density measured at 1 V.

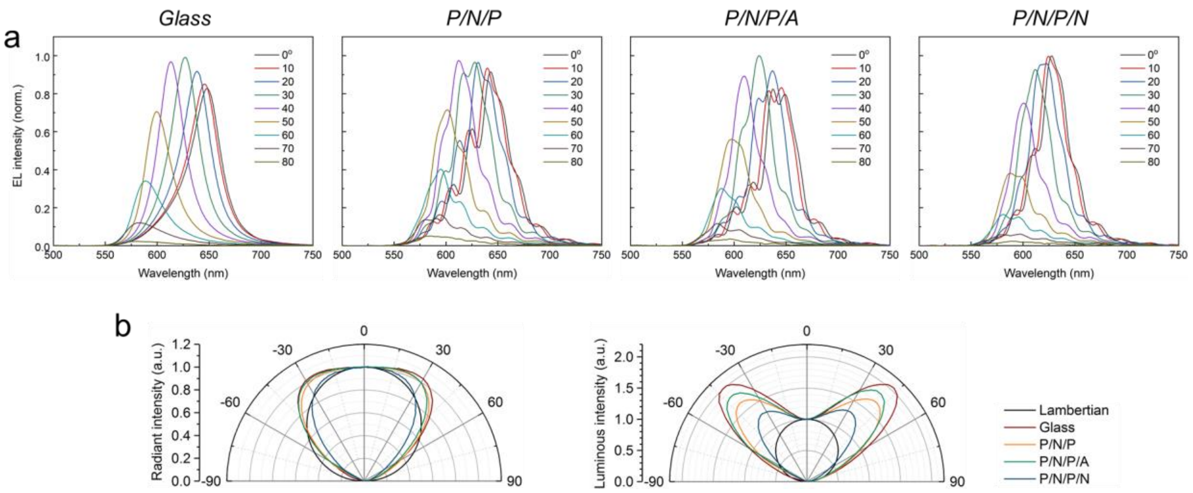

Supplementary Figure 5. Angle-resolved EL spectra and emission intensity. a, Angle-resolved EL spectra of OLEDs with different lower barrier layers and of the reference OLED on glass, each for a set of angles (cf. Fig. 2 in the main manuscript). **b**, Normalized radiant intensity (left) and luminous intensity (right) distributions for same set of devices, calculated from the angle-resolved measurements of EL spectra.

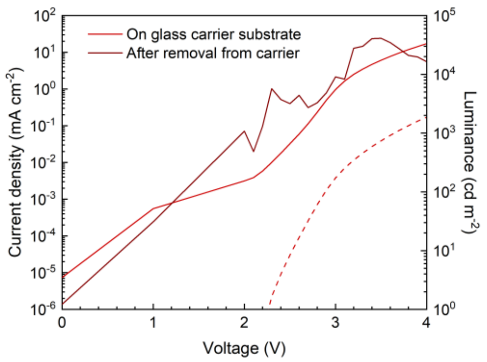

Supplementary Figure 6. Instability of OLEDs built on lower barrier layers formed solely by parylene-C. *j-V-L* characteristics for an OLED deposited on a 10 µm-thick parylene-C film before and after peeling the device from the glass carrier substrate. After removal from carrier, no light emission is observed and current density increases rapidly with voltage, with no diode behaviour observed. This indicates that on its own parylene-C is not able to protect OLEDs sufficiently.

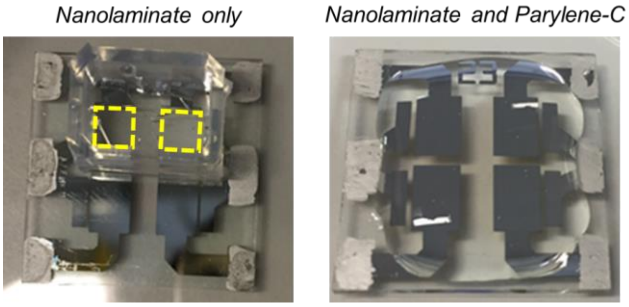

Supplementary Figure 7. Comparing barrier stability in aqueous environment for the nanolaminate on its own versus the hybrid barrier. OLEDs with a top encapsulation layer of nanolaminate (left; total thickness, 150 nm) and hybrid barrier of nanolaminate and parylene-C layers (right) were exposed to cell culture medium (Neurobasal™-A). The former delaminated immediately when immersed into the liquid (the original position of the two delaminated OLED pixels is indicated by dashed yellow lines) while the latter showed no visual change over time. For the nanolaminate-only device, a silicone chamber (ibidi) was attached to confine the culture medium to the OLED pixel area and protect the contact pads but this additional measure did not prevent rapid delamination. While the reason for rapid failure of the nanolaminate-only TFE is not fully clear, it has been suggest that Al_2O_3 prepared using ALD reacts with water which in turn may lead to a change in volume relative to the pristine Al_2O_3 film. This volume change then would likely lead to local defects (cracks, pinholes), through which water can quickly penetrate given the low overall thickness of the nanolaminate. As we expect poor mechanical stability for a substrateless device without parylene-C (which would have a total thickness <1 μm), the stability of the nanolaminates-only TFE was not investigated further.

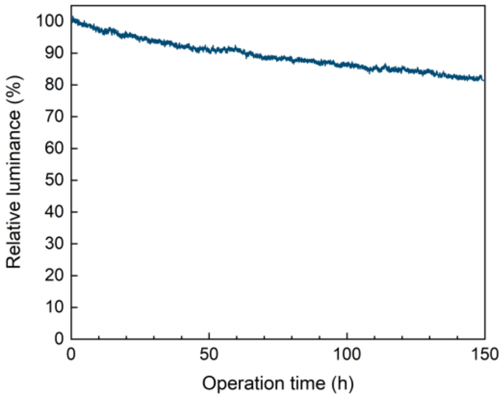

Supplementary Figure 8. Operational lifetime of flexible OLED under constant current. Decay in luminance over time measured under constant current driving (J = 8.4 mA cm^{-2}) for an OLED with a P/N/P/N lower barrier layer. At the current density used for this test, the initial luminance of the device was 2,600 cd m^{-2}.

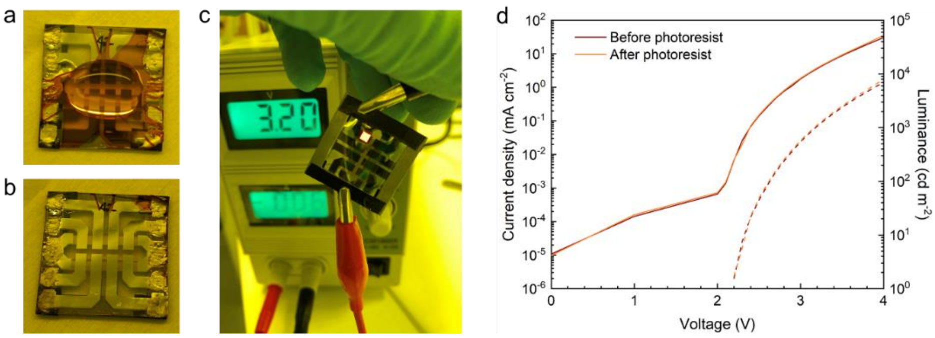

Supplementary Figure 9. Stability of flexible OLEDs to coating with a photoresist. a, Photographs of a flexible OLED attached to a supporting frame after spin-coating a film of photoresist (S1818, Microposit^{TM}) onto the device (with a water droplet on top). **b**, Same device after removing the photoresist by immersion in acetone. **c**, Photograph of OLED after deposition and subsequent removal of photoresist, with an OLED pixel operated. **d**, Comparison of *j*-*V*-*L* characteristics for the same OLED pixel before spin-coating the photoresist and after removing it by immersion in acetone.

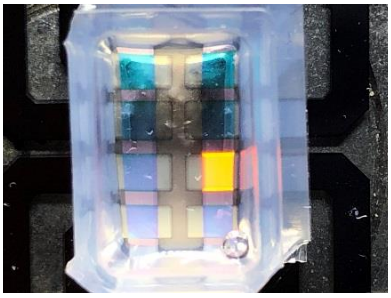

Supplementary Figure 10. Stability test under exposure to cell culture medium. Photograph of an OLED with TFE barrier and a silicone chamber (ibidi) attached that confines culture medium to OLED pixel area and avoids interference of the liquid with the electrical contacts.

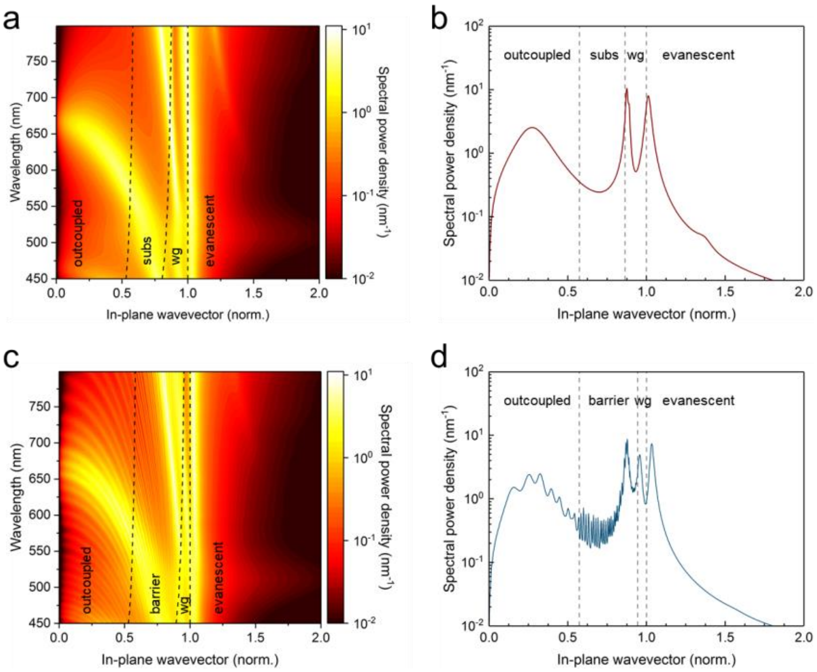

Supplementary Figure 11. Optical simulations for OLEDs on glass and on the flexible TFE barrier. a, **c**, Calculated total power dissipation spectra as function of emission wavelength and the in-plane component of the normalized wavevector. **b**, **d**, Calculated power dissipation spectra at a wavelength of 650 nm as function of the in-plane component of the normalized wavevector. The four regions distinguished by the dashed lines indicate different optical modes, i.e., outcoupled light, substrate (subs) or barrier confined light, waveguided light (wg), and evanescent modes. The panels in the top row (a,b) show results for an OLED on a conventional glass substrate. The bottom row (c,d) summarizes the result for a lower barrier layer with the P/N/P/N structure and thickness described in the main text.

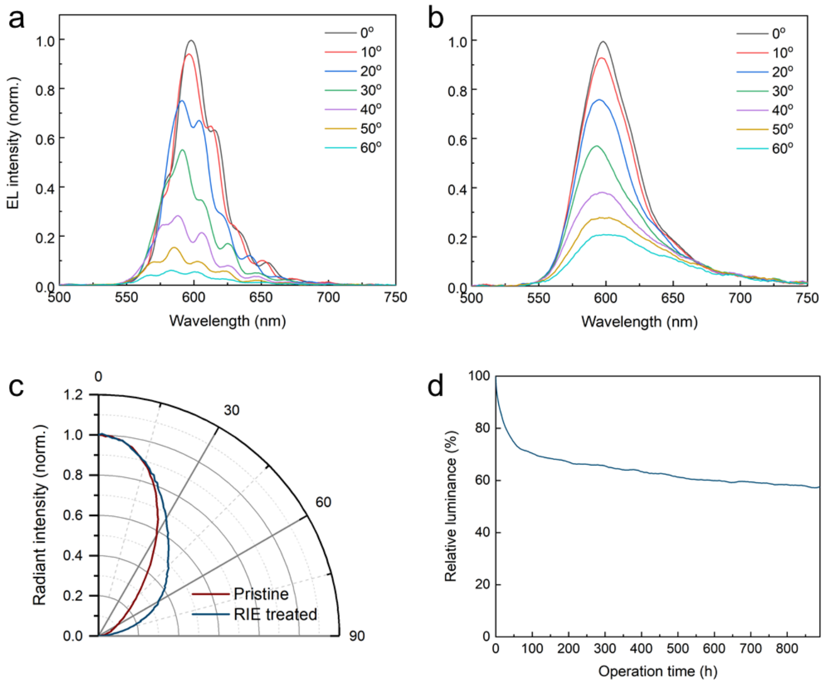

Supplementary Figure 12. Tuning light outcoupling with scattering structure. a, Angle-resolved EL spectra of top-emitting OLEDs with pristine upper TFE barrier. **b**, Angle-resolved EL spectra of top-emitting OLED after roughening upper barrier by RIE. **c**, Comparison of normalized radiant intensity for devices in a and b as calculated from the angle-resolved spectra. **d**, Change in luminance over time under constant current driving (J = 4.38 mA cm^{-2}) for an OLED with an RIE treated upper barrier layer.

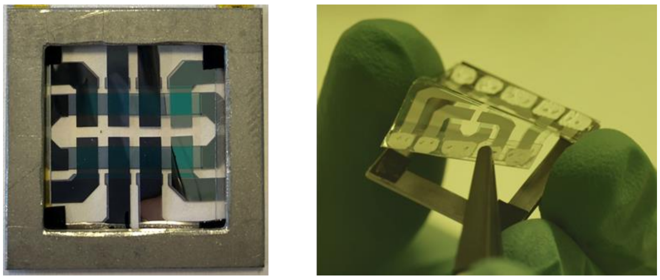

Supplementary Figure 13. Supporting frame. Photographs showing a flexible OLED sample attached to a custom-made supporting frame (left) and the process of detaching a sample from the frame (right).

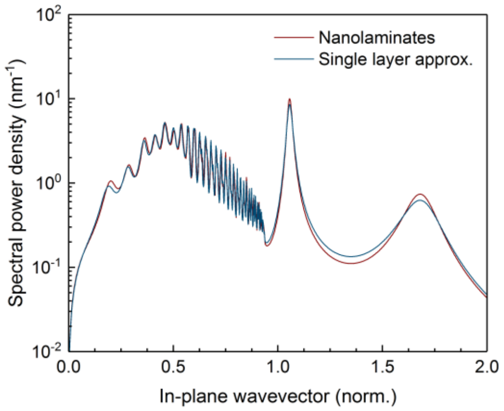

Supplementary Figure 14. Single layer approximation of nanolaminate layers. Comparison of power dissipation spectra simulated with the multilayer model representing the actual experimental structure (red line) and with the single layer approximation described in Methods (blue line).
