# Supplementary material for: A substrateless, flexible, and water-resistant organic light-emitting diode
Source: Nat Commun. 2020 Dec 7;11:6250. doi: 10.1038/s41467-020-20016-3 (PMC7721873; doi:10.1038/s41467-020-20016-3)
Supplement: Supplementary file 3 — Description of Additional Supplementary Files [file 41467_2020_20016_MOESM3_ESM.pdf]

File Name: Supplementary Movie 1

Description: A flexible blue fluorescent OLED with P/N/P/N TFE barriers operated while i) being bent and twisted and ii) being fully immersed in deionized water.

File Name: Supplementary Movie 2

Description: A flexible red phosphorescent OLED with P/N/P/N TFE barriers operated in deionized water.

File Name: Supplementary Movie 3

Description: A flexible red phosphorescent OLED with P/N/P/N TFE barriers operated in acetone. It is seen that the label written on left-hand side of the device dissolves and dissipates between 00:20 and 00:32 in the video.

File Name: Supplementary Movie 4

Description: A flexible red phosphorescent OLED with P/N/P/N TFE barriers operated while being wrapped around a metal rod ($r_b$ = 1.5 mm) and being bent and twisted.

File Name: Supplementary Movie 5

Description: A flexible red phosphorescent OLED with P/N/P/N TFE barriers operated while being folded around the backside of a razor blade ($r_b \approx 0.2$ mm).
